# Insights from *in vivo* micro‐CT analysis: testing the hydraulic vulnerability segmentation in *Acer pseudoplatanus* and *Fagus sylvatica* seedlings

**DOI:** 10.1111/nph.15549

**Published:** 2018-11-22

**Authors:** Adriano Losso, Andreas Bär, Birgit Dämon, Christian Dullin, Andrea Ganthaler, Francesco Petruzzellis, Tadeja Savi, Giuliana Tromba, Andrea Nardini, Stefan Mayr, Barbara Beikircher

**Affiliations:** ^1^ Department of Botany University of Innsbruck Sternwarterstrasse 15 Innsbruck A‐6020 Austria; ^2^ Institute for Diagnostic and Interventional Radiology University Medical Center Goettingen Robert‐Koch‐Straße 40 Göttingen 37075 Germany; ^3^ Max‐Plank‐Institute for Experimental Medicine Hermann‐Rein‐Straße 3 Göttingen 37075 Germany; ^4^ Elettra‐Sincrotrone Trieste Area Science Park Trieste Basovizza 34149 Italy; ^5^ Dipartimento di Scienze della Vita Università di Trieste Via L. Giorgieri 10 Trieste 34127 Italy; ^6^ Department of Crop Sciences Division of Viticulture and Pomology University of Natural Resources and Life Sciences Vienna Konrad Lorenzstrasse 24 Tulln A‐3430 Austria

**Keywords:** beech (*Fagus sylvatica*), embolism, hydraulic vulnerability segmentation, maple (*Acer pseudoplatanus*), seedlings, synchrotron, X‐ray phase contrast micro‐tomography (micro‐CT), xylem

## Abstract

- The seedling stage is the most susceptible one during a tree′s life. Water relations may be crucial for seedlings due to their small roots, limited water buffers and the effects of drought on water transport. Despite obvious relevance, studies on seedling xylem hydraulics are scarce as respective methodical approaches are limited.
- Micro‐CT scans of intact *Acer pseudoplatanus* and *Fagus sylvatica* seedlings dehydrated to different water potentials (Ψ) allowed the simultaneous observation of gas‐filled versus water‐filled conduits and the calculation of percentage loss of conductivity (PLC) in stems, roots and leaves (petioles or main veins). Additionally, anatomical analyses were performed and stem PLC measured with hydraulic techniques.
- In *A. pseudoplatanus*, petioles showed a higher Ψ at 50% PLC (Ψ_50_ −1.13MPa) than stems (−2.51 MPa) and roots (−1.78 MPa). The main leaf veins of *F. sylvatica* had similar Ψ_50_ values (−2.26 MPa) to stems (−2.74 MPa) and roots (−2.75 MPa). In both species, no difference between root and stems was observed. Hydraulic measurements on stems closely matched the micro‐CT based PLC calculations.
- Micro‐CT analyses indicated a species‐specific hydraulic architecture. Vulnerability segmentation, enabling a disconnection of the hydraulic pathway upon drought, was observed in *A. pseudoplatanus* but not in the especially shade‐tolerant *F. sylvatica*. Hydraulic patterns could partly be related to xylem anatomical traits.

## Introduction

Trees can live for hundreds of years, sometimes facing and resisting very harsh environmental conditions during their life span. Yet, the most critical and threatening stages in a tree's life can be tracked back to the very few weeks after seed germination. At the seedling stage, the plant relies upon reserves stored in the seed, until cotyledons unfold and start to perform active photosynthesis. Seedlings and the following juvenile stages are at a very high risk of death (e.g. Fenner, 1987; Larcher, 2003; Smith *et al*., 2003; Johnson *et al*., 2011), because productivity and reserves are small, investments in growing organs have to be perfectly balanced and respective sensitivity to many biotic and abiotic stress factors is high. Water relations are key issues during early ontogeny as root systems are small and shallow, water supply relies on upper soil layers, which are easily exposed to dehydration, and internal water buffers are limited. Despite the small size and relatively short transport distances, multiple studies have indicated that transport hydraulic efficiency and safety of seedlings play a central role in plant survival, just like in adult trees (e.g. Grulke & Retzlaff, 2001; Rice *et al*., 2004; Domec *et al*., 2009). Moreover, the increasing frequency and intensity of drought events (e.g. Sperry & Love, 2015) are limiting the establishment and survival of plants, as seedlings play a critical role in tree population dynamics and shifts in species distributions under climate change (Ibanez *et al*., 2007; Vanderwel *et al*., 2013). Knowledge on seedling hydraulics, therefore, is relevant for various fields such as forestry or nature conservation.

Root‐to‐leaf water transport is necessary to compensate transpirational water losses (cohesion‐tension theory; Boehm, 1893; Dixon & Joly, 1894; Steudle, 2001) and unavoidable generates a water potential (Ψ) gradient along the xylem pathway. The resulting negative hydrostatic pressure in xylem conduits implies the risk of embolism formation and propagation in the xylem (Tyree & Zimmermann, 2002). Embolism causes blockages in xylem conduits that, in turn, reduce plant hydraulic conductance, limit photosynthesis and can even lead to plant death (e.g. Brodribb & Cochard, 2009). Embolism can result from drought stress, when low Ψ cause the aspiration of gaseous bubbles into xylem conduits from adjacent gas‐filled compartments via the pits (air seeding; Tyree & Zimmermann, 2002; see also Choat *et al*., 2015, 2016). Low Ψ is also responsible for embolism formation when plants are exposed to freeze−thaw cycles (e.g. Pittermann & Sperry, 2003; Mayr & Sperry, 2010).

In adult trees, the xylem vulnerability to embolism can differ both between (e.g. Choat *et al*., 2012) and within species (e.g. Beikircher & Mayr, 2009), as well as between organs of specimens (e.g. Tsuda & Tyree, 1997; Beikircher *et al*., 2013; Scholz *et al*., 2014; Johnson *et al*., 2016). It has been suggested that within‐plant variation in vulnerability follows distinct patterns, with distal plant parts, such as leaves or small branches, being more vulnerable to drought‐induced xylem embolism than central and older parts, such as the trunk (hydraulic vulnerability segmentation hypothesis; Tyree & Ewers, 1991; Tyree & Zimmermann, 2002). This would enable drought stressed plants to sacrifice highly vulnerable plant segments by confining embolism in the distal sectors, while keeping the remaining parts hydraulically active and therefore protecting central parts of the water transport system with high carbon investments (e.g. trunk and larger stems). In angiosperms, many studies demonstrated petioles and leaves to be more vulnerable than branches (Tyree *et al*., 1993; Tsuda & Tyree, 1997; Beikircher *et al*., 2013; Scholz *et al*., 2014; Charrier *et al*., 2016; Johnson *et al*., 2016; Wolfe *et al*., 2016). Leaves probably exhibit vulnerable extra‐xylary pathways, which disconnect the xylem from distal water transport under moderate drought stress (Trifilò *et al*., 2016; Scoffoni *et al*., 2017). In some cases, however, trunks were reported to be more vulnerable than branches (e.g. Johnson *et al*., 2016; Rosner *et al*., 2018), and other studies found similar vulnerabilities across organs (e.g. Choat *et al*., 2005; Hao *et al*., 2013). Roots were shown to be more vulnerable than branches in several studies (e.g. Martínez‐Vilalta *et al*., 2002; Maherali *et al*., 2006; Johnson *et al*., 2016). Within‐plant variation in vulnerability has also been reported for conifers (Kavanagh *et al*., 1999; Beikircher & Mayr, 2008; Willson *et al*., 2008; Domec *et al*., 2009; Delzon *et al*., 2010; McCulloh *et al*., 2014; Losso *et al*., 2016; Miller & Johnson, 2017). It is likely that vulnerability segmentation is species‐specific and can show various patterns. These patterns are based on variation in xylem properties (e.g. Hacke *et al*., 2001; Gleason *et al*., 2016), which are known to determine the stability of the hydraulic pathway. Most important, the pit characteristics influences air‐seeding thresholds (Tyree *et al*., 1994; Li *et al*., 2016), and the cell‐wall reinforcement counterbalances maximum tension occurring in xylem conduits (Hacke *et al*., 2001). Most studies on vulnerability segmentation dealt with adult trees (see citations above) and few on shrubs or herbs (e.g. Ganthaler & Mayr, 2015; Nolf *et al*., 2016; Savi *et al*., 2016; Skelton *et al*., 2017), while studies on youngest tree stages (i.e. < 1 yr old) are scarce (Rodriguez‐Dominguez *et al*., 2018). To our knowledge, there are only two studies that directly measured hydraulic vulnerability (Lauenstein *et al*., 2013; Way *et al*., 2013) and none on hydraulic segmentation dealing with plants of an age up to 6 months. This is related to methodical limitations as hydraulic measurements on small plants are difficult. Fortunately, new methods now enable studies on samples of small size, such as the seedlings analysed in the present study.

In past years, a wide variety of experimental techniques has been developed and used for measuring the vulnerability to drought‐induced xylem embolism of different plant organs (Cochard *et al*., 2013). In particular, noninvasive *in vivo* visualisation techniques have recently taken hold in the field of plant hydraulics (e.g. Choat *et al*., 2010; Cochard *et al*., 2015; Jansen *et al*., 2015; Brodribb *et al*., 2016). X‐ray phase contrast micro‐tomography (micro‐CT) is so far the most promising method, as it is nondestructive (but see Petruzzellis *et al*., 2018) and allows *in vivo* observations of conduits status (in terms of water‐ vs air‐filled conduits) and thus to analyse hydraulic integrity and embolism patterns within organs (e.g. Brodersen *et al*., 2013; Choat *et al*., 2015). This technique provides the possibility to visualise at high resolution and quantify xylem embolism in detached branches (e.g. Cochard *et al*., 2015; Choat *et al*., 2016; Knipfer *et al*., 2016; Nardini *et al*., 2017), leaves (Bouche *et al*., 2016; Ryu *et al*., 2016; Scoffoni *et al*., 2017), roots (Cuneo *et al*., 2016) as well as on the main stem of intact plants (Choat *et al*., 2015; Knipfer *et al*., 2017; Nolf *et al*., 2017; Savi *et al*., 2017).

In the present study, we used synchrotron‐based micro‐CT to analyse the vulnerability to drought‐induced xylem embolism of 6‐month‐old *Acer pseudoplatanus* and *Fagus sylvatica* plants (here after called seedlings). The study aimed at real‐time observations of xylem conduits in main organs (stem, roots and leaves) during progressive plant dehydration. Based on the micro‐CT technique, it was not only possible to study intact seedlings but also to compare vulnerability patterns within single plants. We designed an experiment that enabled simultaneous micro‐CT observations at multiple points in intact plants and thus recording within‐plant vulnerability patterns with respect to main roots, stems, petioles or leaf veins. We also compared the theoretical loss of stem hydraulic conductivity (PLC_t_) calculated from micro‐CT observations with classical hydraulic measurements performed on seedling stems, which is important in the view of recent methodical controversies (e.g. Wheeler *et al*., 2013; Trifilò *et al*., 2014; Venturas *et al*., 2015). A comparison between hydraulic and micro‐CT methods on identical plant material has been done in only three recent studies, in which Nardini *et al*. (2017) and Nolf *et al*. (2017) demonstrated agreement between methods, while Savi *et al*. (2017) highlighted possible discrepancies.

We hypothesised that seedlings show pronounced and species‐specific patterns in vulnerability to drought‐induced embolism, with higher hydraulic safety in the main stem compared with leaves and overall small safety in roots. Roots are exposed to overall less negative Ψ as they are situated in the basal part of the soil−plant continuum and accordingly, comparably low hydraulic safety was reported for roots of mature trees (see the third paragraph of the Introduction). Furthermore, we expected similar vulnerability curves from micro‐CT and hydraulic measurements performed using standard protocols. This micro‐CT study should improve our knowledge of xylem hydraulic safety in seedlings, an important aspect in the youngest stage of a tree's life.

## Materials and Methods

All experiments were conducted in September and October 2017 on 6‐month‐old seedlings of *Acer pseudoplatanus* L. and *Fagus sylvatica* L. Seeds (Herzog.Baum, Samen und Pflanzen GmbH, Gmunden, Austria) were sown in small pots (8 cm high and 7 cm wide; to allow an easy handling during experiments). For optimal growing conditions, plants were placed in a glasshouse and constantly irrigated to field capacity (every 2–3 d). At the time of measurements, seedlings were *c*. 15–20 cm tall with a stem diameter of 3–4 mm. Micro‐CT measurements were performed on the following plant organs: stems, main roots (all seedlings had a main root, which was thicker than the others; *c*. 0.5–0.8 mm in diameter), petioles (*A. pseudoplatanus*) and main leaf veins (*F. sylvatica*). Hydraulic measurements were performed only on stems.

### Hydraulic measurements

Vulnerability to drought‐induced xylem embolism was measured hydraulically using the ‘bench dehydration’ technique (Sperry *et al*., 1988; Cochard *et al*., 2013). Fully hydrated seedlings were removed from pots, and roots were carefully rinsed to remove soil residuals. Seedlings were left dehydrating to different water potentials (Ψ) in the laboratory (time intervals ranging between 20 min to 8 h). To allow equilibration of Ψ within plants and obtain accurate Ψ measurements, seedlings were then wrapped in dark plastic bags for 30–45 min before measurement. After dehydration, the apical part of the seedling (3–5 cm including leaves) was used to measure Ψ (Scholander apparatus model 1505D; PMS Instruments, Albany, OR, USA). Out of the central part of the stem, an *c*. 6 cm long sample was cut under water, the bark was removed and the sample trimmed several times with a sharp carving knife to gradually release tension, remove micro‐bubbles (Wheeler *et al*., 2013; Venturas *et al*., 2015) and minimize eventual artefacts due to xylem refilling under rehydration (Trifilò *et al*., 2014). Samples were then connected to a modified Sperry apparatus (Sperry *et al*., 1988; Losso *et al*., 2018) and perfused with distilled and degassed water, filtered at 0.2 μm and containing 0.005% (v/v) Micropur (Katadyn Products, Wallisellen, Switzerland) to prevent microbial growth. The initial hydraulic conductivity (*K*_i_; normalised by xylem cross‐sectional area and sample length) was measured at 4 kPa. *F. sylvatica* samples were then flushed for 10 min at 60 kPa to remove embolism. After flushing, the hydraulic conductivity was measured again. Flushing was repeated until measurements showed no further increase in conductivity to obtain final specific hydraulic conductivity (also normalised by xylem cross‐sectional area and sample length; *K*_s_). All hydraulic measurements were conducted at room temperature (*c*. 21–22°C). Conductivity values were corrected for water viscosity at 20°C, and percent loss of conductivity (PLC) was calculated as:

(Eqn 1) $$\text{PLC}=\left(1-\frac{K_{i}}{K_{s}}\right)\times 100$$

For *A. pseudoplatanus*, direct measurements of PLC were not possible as conductivities progressively decreased upon flushing, indicating conduit plugging. Therefore, PLC was calculated from the conductivity of dehydrated samples (corresponding to *K*_i_ in Eqn (Eqn 1)) vs the conductivity of saturated samples (*K*s in Eqn (Eqn 1); Beikircher & Mayr, 2009). *F. sylvatica* dehydrated very rapidly at Ψ between *c*. −2 to −4 MPa so that only few data points could be measured in this range. Respective vulnerability curves thus show gaps, but determination of vulnerability thresholds was still possible due to sufficient measurements below and above the critical range and to a sufficient number of replicates.

PLC was plotted vs the corresponding Ψ and a Weibull regression curve was fitted to each vulnerability curve (r‐package fit‐PLC, R i386 3.2.5; Duursma & Choat, 2017). From vulnerability plots, we also extracted the thresholds Ψ_12_, Ψ_50_ and Ψ_88_, which refer to Ψ at 12%, 50% and 88% loss of conductivity, respectively (Domec & Gartner, 2001; Choat *et al*., 2012).

### Micro‐CT observations

**Figure 1:**
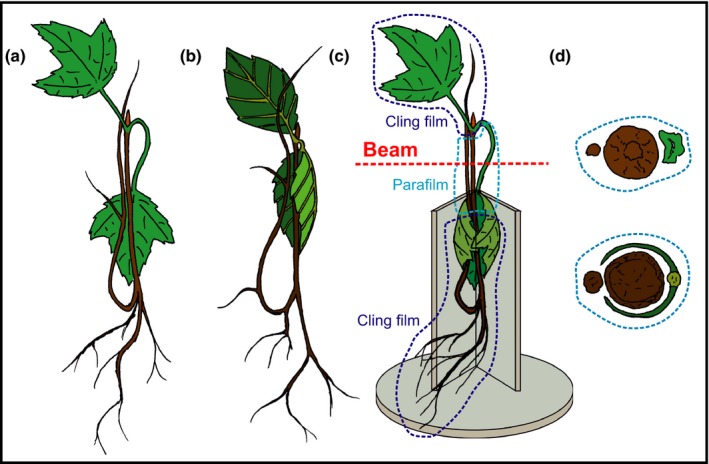
Design of the sample preparation for scanning. The main root and a leaf of *Acer pseudoplatanus* (a) and *Fagus sylvatica* (b) seedlings were bent upwards and downwards, respectively, to bring them at the scanning level of the stem (4–5 cm above the root collar) and wrapped in Parafilm^®^. Remaining parts were wrapped in cling film (to avoid water loss during scanning) and plants placed in a custom‐made sample holder (c). All three organs (stem, root and petiole/main vein) were irradiated and scanned simultaneously (d; *A. pseudoplatanus* and *F. sylvatica* in the upper and lower figure, respectively).

Micro‐CT scans were performed at the SYRMEP beamline of the Elettra Light Source in Trieste, Italy (Tromba *et al*., 2010) using the propagation‐based phase contrast technique. Seedlings were transported to the facility and stored at a shaded field site until preparation for the analyses. Plants were carefully removed from the pots and roots carefully rinsed to remove soil residuals before bench dehydration (as described above in ‘Hydraulic measurements’). To simultaneously observe the functional status of xylem conduits in three different organs (stem, main root and leaf), seedlings were prepared as shown in Fig. 1. Briefly, the main root and a leaf were bent upwards and downwards, respectively, and positioned next to the stem. The section to be scanned (stem, leaf vein or leaf petiole, root) was wrapped in Parafilm®, while the remaining leaves and roots were wrapped in cling film to prevent further dehydration of the sample during scans. For *A. pseudoplatanus*, the petiole was observed. In the case of *F. sylvatica*, due to the short petiole, the main vein of the leaf was observed. Organs were stabilised during scan rotation using a V‐shaped custom‐made sample holder. This allowed an easy and fast positioning of the samples (see Fig. 1), which were fixed to the holder with Terostat putty (Teroson, Heidelberg, Germany). Thanks to the use of this sample holder, we could avoid long exposition to irradiation during the initial sample alignment, thus minimizing eventual X‐ray induced cellular damage (Savi *et al*., 2017; Petruzzellis *et al*., 2018). Overall, sample preparation, initial alignment and scan time (90 s) were performed within 10–15 min. The scanned region of the stem was at *c*. 5–7 cm above the root collar (corresponding to stem sections used for hydraulic measurements), and at a distance of 12 cm from the detector. The field of view was 5 × 5 mm and covered the full cross‐section of the three organs. Two 5‐mm filters of silicon were used to obtain an average X‐ray source energy of 25 keV. The exposure time was set to 100 ms, at an angular step of 2° s^−1^. During the 180° rotation of the sample, 900 projections were acquired. In total, 15 seedlings of *A. pseudoplatanus* and 16 seedlings of *F. sylvatica* at different Ψ were scanned (initial scan). As for hydraulic measurements, in *F. sylvatica*, only few scans were possible between −2 and −4 MPa (see ‘Hydraulic measurements’). After scans, the plant was cut directly above the root collar and Ψ of the upper part (main stem and leaves) was measured with a portable pressure chamber (3005 Plant Water Status Console; Soilmoisture Equipment Corp., Goleta, CA, USA). Stem Ψ was expected to be close to measured Ψ as plants were wrapped in cling film for 20 min at a minimum, which enabled Ψ equilibration. Finally, stem, root and petiole/leaf segments (still wrapped in Parafilm^®^) were cut to *c*. 4 cm length to induce air‐entrance. After 24 h of dehydration, these samples were recut to 1‐cm‐long pieces and arranged in a row along a skewer (four samples per skewer). We then rescanned all samples at the marked position of the first scan to observe fully embolised xylem. Arrangement of several samples in a row enabled time‐efficient consecutive scans by adjusting the stage height (after positioning of the first sample in the beam).

**Figure 2:**
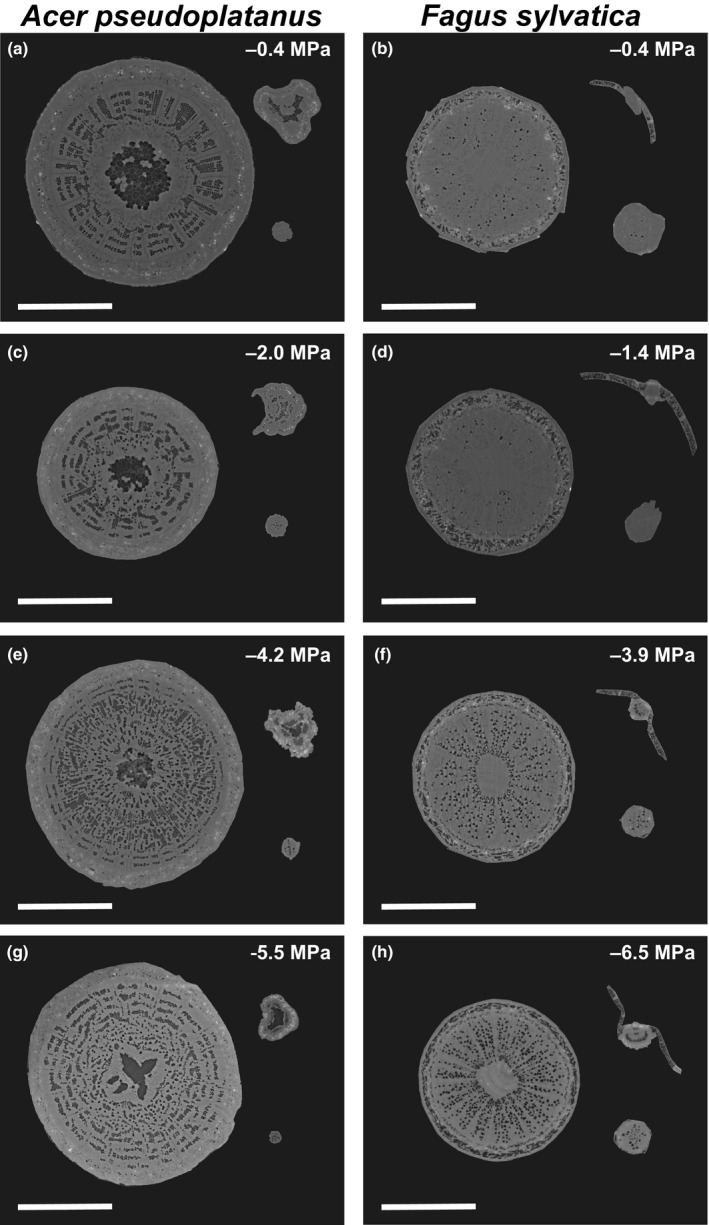
*In vivo* visualization by micro‐CT of xylem embolism in stems (left in each panel), leaves (petiole/main vein; upper right in each panel) and roots (lower right in each panel) in intact *Acer pseudoplatanus* (a, c, e, g) and *Fagus sylvatica* (b, d, f, h) seedlings. Reconstructed cross‐sections show embolised (dark grey) and water‐filled (light grey) xylem conduits at different xylem water potential (MPa). Bars, 1250 μm.

In total, 1400 slices per sample with a pixel size of 2 μm, were reconstructed using the software syrmep TomoProject (STP; Brun *et al*., 2015). In STP, a phase retrieval preprocessing filter (Paganin *et al*., 2002) was applied before the reconstruction using the Filtered Back Projection algorithm. For each sample, one central slice per sample from the initial scan and one from the final scan were analysed using imagej 1.51q software (National Institute of Health, Bethesda, MD, USA). Images were processed to set thresholds and select only air‐filled vessels (black; Fig. 2), whose areas were measured using the ‘Analyse particles’ function. We quantified the vessel density (VD) and the mean diameter (*d*) of each vessel (calculated from its area and assuming circular shape), which was used to calculate the mean hydraulic diameter (*d*_h_) as Σ*d* ^5^/Σ*d* ^4^ (Kolb & Sperry, 1999) per sample and organ. To check for variation in conduit size across plant organs, we also analysed the conduit diameter distribution (2 μm classes). *d* was also used to calculate the theoretical hydraulic conductance (*k*_t_). *k*_t_ is the cumulative hydraulic conductance of all conduits, which was calculated based on a modified Hagen‐Poiseuille equation (e.g. Knipfer *et al*., 2015; Cuneo *et al*., 2016):

(Eqn 2) $$k_{t}=\frac{\pi \rho }{128\mu }\sum_{i=1}^{n}d_{i}^{4}$$

where ρ and μ are the density of the fluid and the viscosity of water, respectively. *k*_t_ was then divided by the cross‐sectional xylem area to obtain the theoretical specific hydraulic conductivity (*K*_st_).

The theoretical percentage loss of hydraulic conductivity (PLC_t_) was calculated by relating the initial theoretical conductivity (*K*_t initial_) to the theoretical conductivity calculated from all visible conduits in the final scan (*K*_t final_):

(Eqn 3) $${\text{PLC}}_{t}=\left(1-\frac{K_{t\;\mathrm{initial}}}{K_{t\;\mathrm{final}}}\right)\times 100$$

whereby *K*_t initial_ was calculated from *K*_t final_ minus *K*_t_ of embolised vessels in the initial scan. PLC_t_ was plotted vs the corresponding Ψ. Exponential sigmoid functions were fitted to each vulnerability curve (see ‘Hydraulic measurements’) and vulnerability thresholds were determined.

### Cell‐wall reinforcement

Samples used for micro‐CT analyses were soaked in an ethanol/glycerol/water solution (1 : 1 : 1, v/v/v) for at least 5 d. Transversal sections were cut with a sliding microtome (Sledge Microtome G.S.L. 1; Schenkung Dapples, Zürich, Switzerland), stained with Etzold FCA mixture (consisting of fuchsine, chrysoidine and astrablue) for 5 min and then rinsed with distilled water. From images captured with a light microscope (Olympus BX41; Olympus Austria, Wien, Austria) connected to a digital camera (ProgRes CT3; Jenoptik, Jena, Germany), we estimated the conduit wall reinforcement by calculating the ‘thickness‐to‐span ratio’ (*t*/*b*)^2^ (Hacke *et al*., 2001) on at least 8–10 conduit pairs per sample. The wall thickness (*t*) and the lumen breadth (*b*) were measured using Imagej 1.51q software. Measurements were made on vessels pairs with a diameter of *d*_h_ ± 2 μm (Hacke & Sperry, 2001; Hacke *et al*., 2001). Values were averaged per plant organ (± SE).

### Statistics

For vulnerability analyses, PLC was plotted vs the corresponding Ψ and a Weibull regression curve was fitted to each vulnerability curve (R package fit‐plc, R i386 3.2.5; Duursma & Choat, 2017). Differences in anatomical (*d*,* d*_h_, VD and (*t*/*b*)^2^) and hydraulic parameters (*K*_st_ and *K*_s_) were tested with a two‐way analysis of variance (ANOVA) followed by Tukey posthoc comparison and Student's *t*‐test, respectively, after testing for normal distribution and homoscedasticity. For vulnerability analyses, differences between techniques and organs were assessed using 95% confidence intervals obtained via bootstrap resampling. Student's *t*‐tests were performed using spss v.24.0 (SPSS Inc., Chicago, IL, USA) at a probability level of 5% while bootstrapping was performed in R studio.

## Results

### Hydraulic measurements and micro‐CT observations

**Figure 3:**
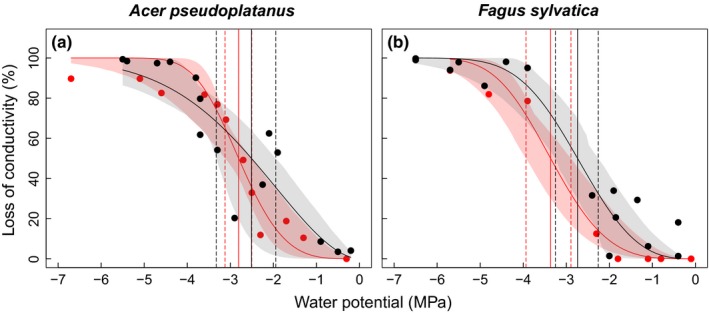
Vulnerability to drought‐induced xylem embolism from hydraulic measurements (red) and micro‐CT observations (black) of stems of *Acer pseudoplatanus* (a) and *Fagus sylvatica* (b) seedlings. Solid vertical lines represent water potential at 50% loss of conductivity (Ψ_50_), dashed vertical lines represent lower and upper confidence intervals for Ψ_50_. Shaded areas represent the 95% bootstrapped confidence interval for fitted curves.

Vulnerability curves obtained with the hydraulic method did not significantly differ between species, though vulnerability thresholds of *A. pseudoplatanus* were overall higher than of *F. sylvatica* (Ψ_50_ −2.81 MPa vs −3.36 MPa; Fig. 3; Table 1). Micro‐CT *in vivo* visualisation of stems confirmed this finding, whereby differences in thresholds were smaller (e.g. Ψ_50_ −2.51 MPa vs −2.74 MPa for *A. pseudoplatanus* and *F. sylvatica*, respectively; Fig. 3; Table 1). Also, vulnerability thresholds did not significantly differ between methods (Fig. 3; Table 1). In both species, calculated stem *K*_st_ was higher than the measured stem hydraulic conductivity *K*_s_ (Table 2).

**Table 1:** Vulnerability to drought‐induced embolism of *Acer pseudoplatanus* and *Fagus sylvatica* stems, roots and leaves obtained with hydraulic measurements and micro‐CT observations

Species	Ψ_12_, MPa	CI 2.5%	CI 97.5%	Ψ_50_, MPa	CI 2.5%	CI 97.5%	Ψ_88_, MPa	CI 2.5%	CI 97.5%	*n*
*A. pseudoplatanus*										
Hydraulic (stem)	−1.83 a	−1.07	−2.17	−2.81 a	−2.54	−3.13	−3.72 a	−3.44	−5.04	13
Micro‐CT (petiole)				−1.13 b*	−0.96	−1.32	−3.01 b	−2.35	−3.54	10
Micro‐CT (stem)	−0.97 a	−0.56	−2.6	−2.51 a	−1.93	−3.33	−4.69 a	−3.78	−5.69	15
Micro‐CT (root)	−1.41 a	−1.39	−2.18	−1.78 a	−1.77	−2.48	−2.08 b	−1.97	−2.7	10
*F. sylvatica*										
Hydraulic (stem)	−2.16 a	−1.37	−2.65	−3.36 a	−2.92	−3.94	−4.52 a	−4.15	−5.27	9
Micro‐CT (leaf main vein)	−1.30 a	−0.67	−3.02	−2.26 a	−1.93	−3.31	−3.25 a	−2.53	−5.15	12
Micro‐CT (stem)	−1.54 a	−0.93	−2.26	−2.74 a	−2.28	−3.24	−4.00 a	−3.09	−5.11	16
Micro‐CT (root)	−1.73 a	−1.16	−2.32	−2.75 a	−2.00	−3.33	−3.74 a	−2.07	−4.77	8

Parameters Ψ_12_, Ψ_50_ and Ψ_88_ correspond to Ψ at 12, 50 and 88% loss of conductivity, respectively, and CI 2.5% and CI 97.5% indicate the confidence interval for each parameter. *n*, the number of samples used for plotting each vulnerability curve. Letters indicate statistically significant differences in the respective parameter within a species, asterisks between species (*P *<* *0.05).

**Table 2:** Mean conduit diameter (*d*), mean vessel hydraulic diameter (*d*_h_), vessel density (VD), cell‐wall reinforcement (*t*/*b*)^2^ and mean theoretical specific hydraulic conductivity (*K*_st_) of roots, stems and leaves, and mean specific hydraulic conductivity (*K*_s_) of stems of *Acer pseudoplatanus* and *Fagus sylvatica* seedlings

Species	Organ	*d* (μm)	*d*_h_ (μm)	VD (*n* mm^−2^)	(*t*/*b*)^2^	*K*_st_ (kg m^−1^ MPa^−1^ s^−1^)	*K*_s_ (kg m^−1^ MPa^−1^ s^−1^)
*A. pseudoplatanus*	Root	11.15 ± 1.13 a*	15.63 ± 0.71 a*	1284.0 ± 2.6 a*	0.083 ± 0.006 a	0.95 ± 0.13 a*	
	Stem	17.24 ± 1.50 b	25.06 ± 1.89 b	211.5 ± 0.8 b*	0.070 ± 0.008 a*	0.95 ± 0.16 a	0.14 ± 0.01**
	Petiole	10.47 ± 0.23 a*	13.96 ± 0.26 a*	908.3 ± 1.1 a	0.063 ± 0.006 a	0.76 ± 0.18 a*	
*F. sylvatica*	Root	14.51 ± 1.44 a	22.65 ± 1.09 a	741.3 ± 2.1 a	0.080 ± 0.009 a	2.01 ± 0.24 a	
	Stem	18.36 ± 0.64 b	23.72 ± 0.67 a	314.4 ± 0.6 b	0.140 ± 0.004 b	1.35 ± 0.13 b	0.34 ± 0.05**
	Main leaf vein	12.01 ± 1.25 a	16.42 ± 0.88 b	2551.3 ± 3.9 c	0.074 ± 0.018 a	2.51 ± 0.60 a	

Letters indicate statistically significant differences in the respective parameter within a species, asterisks between species (*P *<* *0.05). Double asterisks indicate significant differences between *K*_st_ and *K*_s_ within a species (*P *<* *0.05). Mean ± SE.

**Figure 4:**
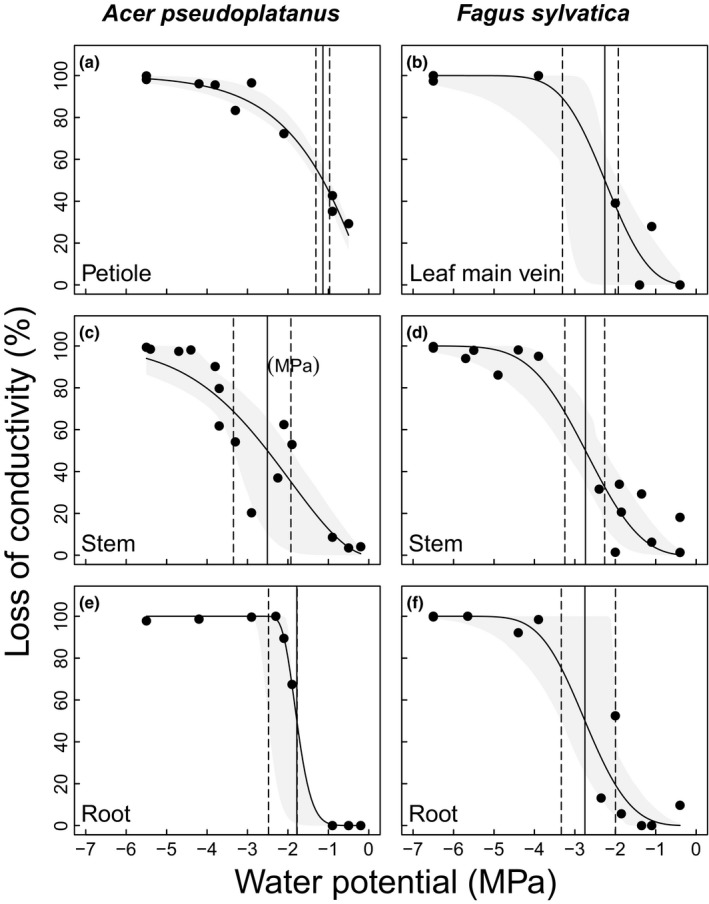
Vulnerability to drought‐induced xylem embolism of micro‐CT observations of leaves (a, b), stems (c, d) and roots (e, f) of *Acer pseudoplatanus* and *Fagus sylvatica* seedlings. Solid vertical lines represent water potential at 50% loss of conductivity (Ψ_50_), dashed vertical lines represent lower and upper confidence intervals for Ψ_50_. Grey shaded areas represent the 95% bootstrapped confidence interval for fitted curves. Stem curves are also shown in Fig. 3.

Micro‐CT also revealed similar Ψ_50_ in roots of study species (Table 1). In *A. pseudoplatanus*,* in vivo* visualisation indicated petioles (Ψ_50_ −1.13 MPa) to be more vulnerable than both roots and stems (Fig. 4; Table 1), while roots and stems did not differ in Ψ_50_. However, roots exhibited significantly higher Ψ_88_ than stems (−2.08 MPa vs −4.69 MPa) with a steep increase in PLC upon decreasing Ψ (Fig. 4e). In petioles, Ψ_12_ could not be determined, because data points at high Ψ were missing. Petiole *K*_st_ was lower than root and stem *K*_st_, which showed similar values (Table 2). In *F. sylvatica*, vulnerability to drought‐induced xylem embolism of stems, roots and leaf veins did not differ (Fig. 4b,d,f; Table 1). Main leaf vein *K*_st_ was higher than root *K*_st_, which was higher than stem *K*_st_ (Table 2).

### Anatomical parameters

**Figure 5:**
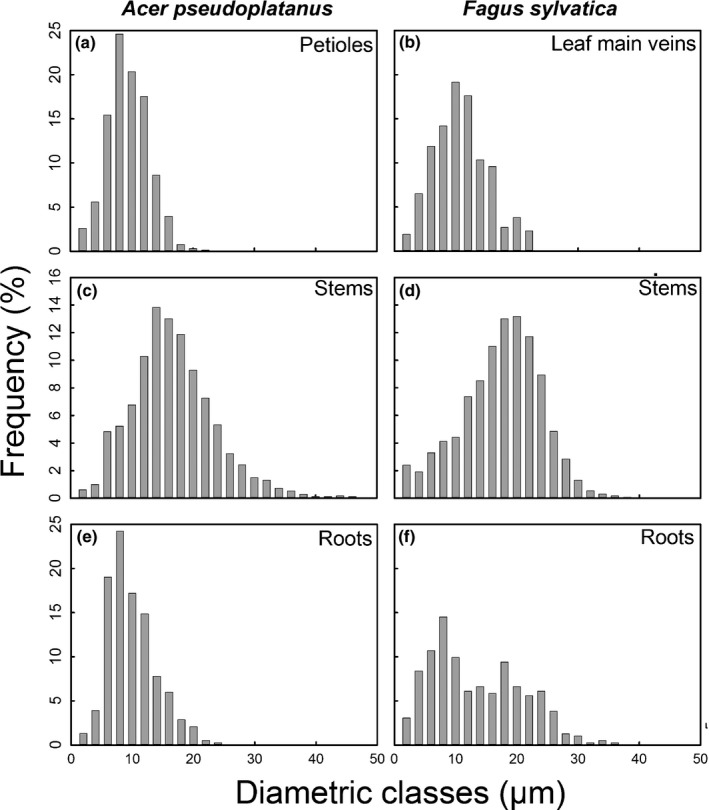
Distribution of conduit diameters (2 μm classes) of stems (a, b), roots (c, d) and leaves (e, f) of *Acer pseudoplatanus* (a, c, e) and *Fagus sylvatica* (b, d, f) seedlings.

Both species showed similar stem *d* and *d*_h_ (Table 2), whereby in *A. pseudoplatanus* the size of most frequent conduits was smaller and a fraction of large diameter conduits (> 38 μm) present, which were missing in *F. sylvatica* (Fig. 5). In *A. pseudoplatanus*, leaf and root xylem showed significantly smaller *d* and *d*_h_ values (Table 2) than in *F. sylvatica*.

In *A. pseudoplatanus*, pronounced differences in the number of larger conduits (> 20 μm) were observed between organs (Fig. 5a,c,e). In petioles, *d* and *d*_h_ were smaller than in roots, while largest *d* and *d*_h_ were found in stems (Table 2; see also Fig. 5). In *F. sylvatica*,* d*_h_ of roots (23.72 ± 0.67 μm) and stems (22.65 ± 1.09 μm) was similar (see Table 2) while *d* and diametric classes distribution differed (Fig. 5d,f; Table 2). Major leaf veins of *F. sylvatica* showed smaller *d* and *d*_h_ (Table 2; see also Fig. 5) than roots and stems.

Both species showed low stem VD when compared with roots and leaf veins/petioles (Table 2). In *A. pseudoplatanus*, root and petiole VD did not differ, while leaf veins had a significantly higher VD than roots in *F. sylvatica*. Cell‐wall reinforcement (*t*/*b*)^2^ showed minor variation in *A. pseudoplatanus*. In *F. sylvatica*, stems exhibited higher (*t*/*b*)^2^ values than both roots and leaf (Table 2).

## Discussion

Based on the micro‐CT technique, xylem resistance to drought‐induced embolism in seedlings of two angiosperm species could be studied *in vivo*. The experimental design enabled the analysis of vulnerabilities in different organs of individual plants. Micro‐CT observations demonstrated within‐plant variation in the vulnerabilities of one out of two study species and thus species‐specificity in the hydraulic architecture of seedlings. Moreover, comparison of micro‐CT observations and hydraulic measurements indicated agreement between methods.

Micro‐CT scans enabled the analysis of within‐plant patterns in hydraulic traits at small scale. In the present study, this technique was used to compare the hydraulic vulnerability of stem, roots and leaves within intact seedlings, which had an age of only few months, while previous studies dealt with either saplings or detached organs of adult trees (e.g. Choat *et al*., 2015, 2016; Cochard *et al*., 2015; Bouche *et al*., 2016; Cuneo *et al*., 2016; Knipfer *et al*., 2016, 2017; Ryu *et al*., 2016; Nardini *et al*., 2017; Nolf *et al*., 2017; Scoffoni *et al*., 2017). Interestingly, the mean vulnerability of seedling stems was lower than that of branches from adult trees of *A. pseudoplatanus* (Ψ_50_ −1.60 and −2.2 MPa from Tissier *et al*., 2004 and Lens *et al*., 2011; respectively). In 2‐yr‐old saplings, Lübbe *et al*. (2017) also found lower Ψ_50_ (*c*. −3.70 MPa) compared with branches of adult trees (Tissier *et al*., 2004; Lens *et al*., 2011). In contrast, *F. sylvatica* seedlings showed a stem Ψ_50_ similar to adult trees and to 2‐yr‐old saplings (Lübbe *et al*., 2017; Bär *et al*., 2018). This indicates that early ontogenetic stages of *A. pseudoplatanus* are probably better protected against drought‐induced xylem dysfunction than adult trees, which may be necessary to counterbalance the small root system (see ‘Introduction’). For *F. sylvatica* seedlings, this aspect may be less relevant due to this species’ overall lower hydraulic vulnerability and its shade tolerance. *F. sylvatica* is a very shade‐tolerant and heavy shade‐casting species (Petritan *et al*., 2007) and may be better protected in the understory from risky transpirational losses. Though, the recorded Ψ_88_ was less negative than in 2‐yr‐old saplings in both species (−6.00 and −4.80 MPa for *A. pseudoplatanus* and *F. sylvatica*, respectively; Lübbe *et al*., 2017). Intense drought events thus might produce significantly larger impacts and mortality in seedlings. However, comparison of vulnerability thresholds of our seedlings with mature trees analysed in previous studies have to be taken with caution as different methods and techniques were used.

Petioles of *A. pesudoplatanus* (Fig. 4a) were found to be more vulnerable than both stems and roots, while in *F. sylvatica*, organs exhibited similar vulnerabilities (Fig 3d,f,h; Table 1). It should be noticed that leaf vulnerability analyses considered only xylary pathways (in petioles or main veins) but not extra‐xylary components (Trifilò *et al*., 2016; Scoffoni *et al*., 2017). Recent studies on angiosperm species (Klepsch *et al*., 2018; Wason *et al*., 2018) also reported leaf xylem to be similarly resistant to embolism compared with other organs except for *Acer rubrum* (Wason *et al*., 2018), which exhibited more vulnerable petioles than stems. Interestingly, in roots of *A. pseudoplatanus*, Ψ_12_ and Ψ_50_ were similar to stems, while Ψ_88_ was less negative (Fig. 4c, e; Table 1). This indicates resistance of roots to moderate drought, which likely can occur in upper soil layers. Rodriguez‐Dominguez *et al*. (2018) found roots of *Olea europaea* saplings to have more resistant xylem than stems and leaves, and Tsuda & Tyree (1997) reported higher hydraulic safety in roots compared with stems in *A. saccharinum* saplings. In contrast, several studies indicated fine roots to exhibit low hydraulic safety and thus to act as hydraulic ‘fuses’ (Jackson *et al*., 2000; Cuneo *et al*., 2016). Though, the present micro‐CT study focused on the seedlings’ main root, which (although similar in size) may differ from fine roots.

During the dehydration process, leaves of both species under study started to wilt below distinct Ψ. This was particularly pronounced in *A. pseudoplatanus*, which suffered severe wilting at Ψ of *c*. −1 to −1.5 MPa. Wilting thus happened before Ψ_50_ was reached, even in *A. pseudoplatanus*, whose petioles showed the highest vulnerability in this study. As suggested by other authors (Tyree *et al*., 1993; Pivovaroff *et al*., 2014; Savi *et al*., 2016; Wolfe *et al*., 2016), leaf wilting and shedding might play an important role under drought stress by reducing transpiration and water loss. Embolism formation in the leaf petiole and/or leaf veins is expected to further disconnect the main stem from leaves and thus delay the decrease of stem Ψ and the risk of xylem dysfunction. Under drought, a timely onset of these protective mechanisms may be essential to guarantee survival in young seedlings.

Anatomical parameters (i.e. *d*,* d*_h_, VD and (*t*/*b*)^2^) were overall similar between organs (Table 2) and hardly reflected observed variations in hydraulic vulnerability. In *A. pseudoplatanus*, no difference in conduit size (*d* and *d*_h_) or VD between petioles and roots were observed, while the stem exhibited wider conduits but lower VD. Similar values of (*t*/*b*)^2^, which is related to the hydraulic vulnerability (e.g. Hacke & Sperry, 2001; Hacke *et al*., 2001), were recorded across organs. In *A. rubrum*, Wason *et al*. (2018) reported similar hydraulic safety across organs despite differences in conduit diameters. In *F. sylvatica*, despite the absence of differences in hydraulic vulnerability, stems exhibited wider conduits, lower VD and lower (*t*/*b*)^2^ than roots and leaf main veins. These veins showed the highest VD values as conduits were smaller in size and grouped in bundles. We suppose that mechanical demands substantially influenced the measured anatomical parameters and masked possible hydraulic structure‐function relationships. Also, the properties of pits, which are central structures with respect to drought‐induced xylem dysfunction (e.g. Cochard *et al*., 2009; Lens *et al*., 2011; Li *et al*., 2016), were not considered in our study but relevant as indicated by the differences between *K*_st_ calculated form micro‐CT images and hydraulically measured *K*_s_. More studies, ideally based on micro‐CT analysis at higher resolution, would be desirable to investigate the relation between pit characteristics and hydraulic vulnerability segmentation in more detail.

In both species under study, stem vulnerability curves based on hydraulic measurements were similar to those obtained with micro‐CT observations. These results agree with previous studies (e.g. Nardini *et al*., 2017; Nolf *et al*., 2017) and indicate that, when appropriate precaution is taken, ‘cutting artefacts’ (Wheeler *et al*., 2013) do not bias hydraulic measurements by causing an overestimation of vulnerability. The Ψ_50_ of hydraulic curves was even more negative than Ψ_50_ obtained with micro‐CT (Fig. 3; Table 1), as also reported by Savi *et al*. (2017) for young sunflower stems. This difference between methods can be attributed to three main methodological limitations: (1) Micro‐CT observations can lead to an overestimation of conductivity, when conduits are water filled but do not contribute to sap flow. This may be the case when conduits are not connected to adjacent ones (e.g. due to immature conduits). (2) Micro‐CT only allows an estimation of conductivities based on conduit diameter, and calculations do neither include pit resistances nor resistances caused by the cell walls. (3) Hydraulic measurements on small plant material are difficult and may not be highly accurate due to e.g. conduit clogging, cut open vessels, accidental removal of embolism during sample preparation and gas exsolution. Accordingly, hydraulic vulnerability measurements never showed a PLC higher than 95%, while in micro‐CT observations, 100% PLC were reached already at −4.5 MPa (see Fig. 3). In a recent study, Savi *et al*. (2017) suggested that higher rates of PLC in micro‐CT based vulnerability curves might be caused by the exposition of samples to heat and/or X‐ray absorption during sample alignment and scanning. However, in our study, samples were not exposed to irradiation during the initial alignment and the scan time was rather short (90 s) compared with other studies (Cochard *et al*., 2015; Choat *et al*., 2016; Knipfer *et al*., 2016; Ryu *et al*., 2016; Nardini *et al*., 2017; Nolf *et al*., 2017).

Further analyses on larger species pools and on wider spatial scales (e.g. including fine roots), as well as a combination with other important hydraulic and physiological traits (e.g. water storage, leaf shedding) will be essential to better understand water relations of trees at early ontogenetic stages. Improved knowledge on seedling hydraulics will be an important base to optimise afforestation strategies and future management under climate change projections of progressively warmer and drier conditions.

## Author contributions

BB, SM and AL planned and designed the present study. AL performed hydraulic experiments. AL, AB, BD, CD, AG, FP, TS, GT, SM and AN were involved in micro‐CT observations. Important technical support was given by GT, FP and CD. AL and AG made the image reconstruction. AL, AB, AG, BB, SM and AN performed data analyses and interpretation. The manuscript was prepared by AL, SM and BB with contributions from all other authors.
